# Questionnaire Validation of Colorectal Cancer Literacy Scale among Thai People in Northeastern Thailand

**DOI:** 10.31557/APJCP.2019.20.2.645

**Published:** 2019

**Authors:** Chontira Kawthaisong, Supannee Promthet, Supot Kamsa-Ard, Rujira Duangsong

**Affiliations:** 1 *Doctor of Public Health Program,*; 3 *Department of Epidemiology and Biostatistics, *; 4 *Department of Public Health Administration Health Promotion Nutrition, Faculty of Public Health, *; 2 *ASEAN Cancer Epidemiology and Prevention Research Group, Khon Kaen University, Thailand.*

**Keywords:** Colorectal cancer screening literacy, instrument development, validation, Thai people

## Abstract

**Background:**

Colorectal cancer is an important public health problem worldwide. Although progress in screening and treatment has considerably improved the prognosis in the developed world, in developing countries colorectal cancer mortality rate remains relatively high. Colorectal cancer screening literacy is an important initial step in overcoming this problem. Development of a validated assessment instrument is therefore important for implementation of appropriate health education programs to facilitate early detection.

**Objectives:**

This study focused on generation and validation of a colorectal cancer screening literacy scale for Thai people in northeastern Thailand.

**Methods:**

This methodological study was carried out in two phases: (1) literature reviews and semi-structured interviews were used to select items, then the content and face validity were checked; and (2) a confirmatory factor analysis (CFA) was conducted to test construct validity and reliability. A self-administered questionnaire was used to collect data from Thai people aged 50-65 in June 2017.

**Results:**

For the total of 400 participants who responded (response rate 100 %), the age ranged from 50 to 65 years old (mean = 57.3, SD = 4.616). The colorectal cancer screening literacy scale was designed to include 6 domains and it was shown to have good internal consistency, and CFA demonstrated the model to fit data adequately (Chi-squared/degree of freedom = 1.079, p = 0.061, CFI = 1.00, GFI = 0.93, AGFI = 0.91, RMSEA = 0.014 and SRMR = 0.036). The final version of its, consisting of 57 items across the 6 domains covering key aspects of colorectal cancer screening literacy, demonstrated good psychometric properties for this population.

**Conclusions:**

Use of the colorectal cancer screening literacy scale in Thai people could lead to improved educational programs for optimizing colorectal cancer screening.

## Introduction

Colorectal cancer (CRC) is the third most common cause of cancer death in men and the second in women worldwide (World Health Organization, 2015). In Thailand, it is the third cause of cancer mortality in men and the fourth in women (National Cancer Institute of Thailand, 2014). Since advanced CRC can be prevented by routine screening and early detection with a removal of lesions in early stages, reducing morbidity and mortality, many countries have implemented national screening programs (National Health Service (England), 2015; Bradley et al., 2015; Tinmouth et al., 2011). With establishment of the idea of individual autonomy when making decisions about screening, it has increasingly been accepted that screening programs should focus on an informed decision-making approach. The preventable characteristics of this cancer and the fact that incidence rates rise after age 50 has led to age-based suggestions for CRC routine screening. Inadequate health literacy is a significant factor associated with low CRC screening rates (Kobayashi et al., 2014). Health literacy is defined as “the capacity to obtain, process, and understand basic health information and services to make appropriate decisions” (World Health Organization, 2009). 

Clinical guidelines recommend that adults aged 50–75 should undergo regular CRC screening, but the existence of several available test options means that people must be fully informed about possible concerns (Bibbins-Domingo et al., 2016; Sepucha et al., 2004). In many places people have been shown to have important knowledge gaps about CRCs (Hoffman et al., 2010; Dolan et al., 2004; Shokar et al., 2010). Factors that are significant when considering what screening to undergo include the invasiveness of modality chosen and the necessity for, preparation (Dolan et al., 2004; Shokar et al., 2010; Dam et al., 2010). There is a need for a comprehensive assessment of decision-making capacity. 

A recent study into facilitating informed decision-making for the Australian colorectal screening program demonstrated a negative impact of limited health literacy when assimilating complex information about cancer control and prevention (Smith et al., 2008). Improvement in health literacy is clearly a high priority (Ojinnaka et al., 2015; Arnold et al., 2012; Kutner et al., 2006), with identification of barriers (Khankari et al., 2007). Health care provider communication skills (Miller et al., 2007; White et al., 2008) and frequency of CRCs recommendations (Miller et al., 2007) are areas requiring assessment for interactive and critical health literacy (Van der Heide et al., 2015). Health education is needed to enhance individual’s knowledge, understanding and ability to act (Nutbeam, 2008), with a focus on perceived susceptibility and health-promoting behavior (Hoffman et al., 2010; Dolan et al., 2004; Shokar et al., 2010; Miller et al., 2007; Peterson et al., 2007; Wolf et al., 2001; Agre et al., 2006). The present study was therefore undertaken to develop and validate an instrument to assess colorectal cancer screening literacy among Thai people in northeastern Thailand.

## Materials and Methods

*Sample and data collection*

Our study was carried out in the general population aged 50 to 65 years in Northeastern Thailand in June, 2017. Sample size calculation for factor analysis was based on an appraisal to create construct validity (Comrey and Lee, 1992). Communities from rural and urban areas of Khon Kaen and Ubon Ratchathani provinces underwent stratified random sampling for participant selection. Permission to collect data was obtained with the help of heads of each community. A total sample of 400 Thai people was enrolled in the areas based on two criteria (locality and age groups) using convenience sampling. All participants were provided with detailed information by the researchers and informed consent was provided before data collection. People diagnosed with colorectal cancer or unlettered in the Thai language were excluded. The study protocol was approved by the ethics committees of Khon Kaen University (HE 592237).

*Instrument development*

A comprehensive literature review was conducted along with a semi-structured interview of a small sample of Thai people to assess potential items for the colorectal cancer screening literacy scale (CRC-LS). Literature searches were made using electronic journal databases for articles written in English and published between 2001 and 2017. Semi-structured interviews were carried out with a sample of 15 Thai people and 6 experts including a behavioral health specialist, an oncologist and a nurse to explore the level of colorectal cancer literacy. This process revealed 84 items across six domains which were subsequently assessed by six research staff with extensive experience in the colorectal cancer field, behavioral sciences and psychosocial constructs. Some items were revised after this assessment and then a pilot study was conducted to evaluate face validity.

Apart from 12 variables related to demographic characteristics, the final version of the self-administered questionnaire included 57 items relating to colorectal cancer literacy, distributed among six domains of literacy: 1) Knowledge and understanding of colorectal cancer screening, established risk factors, signs and symptoms; 2) Accessibility of service and information about colorectal cancer screening and personal barriers, including emotional, practical and service delivery; 3) Communication skills allowing active participation in everyday activities, to extract information and derive meaning and apply to changing circumstances; 4) Self-management of colorectal cancer screening; 5) Media literacy, exploring personal skills to analyze information critically and use it to exert greater control over life events and situations; and 6) Decision skills, considering attitudes to personal participation in colorectal cancer screening. 

**Table 1:** Demographic Characteristics of Participants

Characteristics	n=400	%
Gender		
Male	173	43.2
Female	227	56.8
Age group		
Adults (50-59 y)	264	66
Elderly (60-65 y)	136	34
Mean (SD)	57.38 (4.616)	
(Min : Max)	(50 : 65)	
Education level		
Primary school	343	85.8
Junior high school	30	7.5
Senior high school	23	5.7
Bachelor degree	3	0.7
Higher than Bachelor degree	1	0.3
Occupation		
Agriculture	340	85
Government	5	1.3
Trader	23	5.7
Laborer	27	6.7
Other	5	1.3
Monthly income		
< 10,001 THB	388	97.0
10,001 to 20,000 THB	8	2.0
20,000 to 30,000 THB	1	0.2
≥ 30,000 THB	3	0.8
Mean (SD)	3,405.50 (4529.593)	
(Min : Max)	(0 : 45,000)	
Province		
Khon Kaen	200	50.0
Ubon Ratchathani	200	50.0
Locality		
Rural	200	50.0
Urban	200	50.0
Family history of cancer		
Yes	63	15.7
No	337	84.3
Smoking history		
Yes	91	22.8
No	309	77.2
Alcohol consumption history		
Yes	142	35.5
No	258	64.5
Body mass index (BMI) kg/m2		
≤ 18.5	21	5.3
18.5 to 22.9	158	39.5
23.00 to 24.9	97	24.2
≥ 25	124	31.0
Colorectal cancer screening history		
Yes	19	4.8
No	381	95.2

**Table 2:** Standardized Loading Factors for Confirmatory Factor Analysis of the CRC-LS

Domains	Knowledge	Accessibility	Communication skill	Self- management	Media literacy	Decision skills
Knowledge and understanding of colorectal cancer (23 items)	0.36 to 0.66					
Accessibility of information and service about colorectal cancer screening (6 items)		0.01 to 0.90				
Communication skill with health care providers (11 items)			0.59 to 0.82			
Self-management in the prevention of colorectal cancer (7 items)				0.61 to 0.87		
Media literacy of colorectal cancer screening information (5 items)					0.68 to 0.91	
Decision skills for Colorectal Cancer Screening (5 items)						-0.52 to -0.87

**Table 3:** Statistics Fit Using Confirmatory Factor Analysis of the CRC-LS

Domains	b (SE)	R2	χ2/df	p-value	RMSEA^a^	SRMR^b^	CFI^c^	GFI^d^	AGFI^e^
Knowledge and understanding of colorectal cancer screening	0.33 **(0.07)	0.11	1.012	0.445	0.005	0.032	1	0.97	0.93
Accessibility of information and service about colorectal cancer screening	0.63 **(0.06)	0.4	1.323	0.178	0.028	0.026	1	0.99	0.96
Communication skill with health care providers	0.91 **(0.06)	0.84	1.098	0.345	0.016	0.013	1	0.99	0.97
Self-management in the prevention of colorectal cancer	0.79 **(0.06)	0.63	1.033	0.41	0.009	0.016	1	0.99	0.98
Media literacy for colorectal cancer screening information	0.94 **(0.06)	0.88	1.79	0.167	0.045	0.012	1	1	0.97
Decision skills for colorectal cancer screening	0.9 **(0.07)	0.82	1.465	0.164	0.034	0.027	1	0.99	0.97

^a^, root mean square error of approximation; ^b^, standardized root mean square residual; ^c^, comparative fit index; ^d^, goodness of fit index; ^e^, adjusted goodness of fit index; **, p<0.01.

**Table 4:** Results of Second-Order CFA Model of the CRC-LS

Instrument	χ2/df	p-value	RMSEA^a^	SRMRb	CFI^c^	GFI^d^	AGFI^e^
The CRC-LS-I (69 items)	2.604	0	0.063	0.093	1	0.75	0.66
The CRC-LS-II (57 items)	1.079	0.061	0.014	0.036	1	0.93	0.91

^a^, root mean square error of approximation; ^b^, standardized root mean square residual; ^c^, comparative fit index; ^d^, goodness of fit index; ^e^, adjusted goodness of fit index ; **, p<0.01.

**Table 5:** Inter-Scale Correlations (Pearson’s Correlation)

Domains	Knowledge and understanding of colorectal cancer screening	Accessibility of information and service about colorectal cancer screening	Communication skill with health care providers	Self-management in the prevention of colorectal cancer	Media literacy of colorectal cancer screening information	Decision skills for Colorectal Cancer Screening
Knowledge and understanding of colorectal cancer screening	1	0.15	0.23	0.21	0.24	-0.2
Accessibility of information and service about colorectal cancer screening	0.15	1	0.55	0.48	0.56	-0.47
Communication skill with health care providers	0.23	0.55	1	0.76	0.89	-0.74
Self-management in the prevention of colorectal cancer	0.21	0.48	0.76	1	0.78	-0.66
Media literacy of colorectal cancer screening information	0.24	0.56	0.89	0.78	1	-0.76
Decision skills for colorectal cancer screening	-0.2	-0.47	-0.74	-0.66	-0.76	1

*Statistical analysis*

Demographic characteristics of the participants were described using frequency and percentage values for categorical data and mean and standard deviation for continuous data. Given that speculated constructs were stated in priority, confirmatory factor analysis (CFA) was used. Items were first managed with a standardization sample and the number of items set in accordance with the protocol. Re-management with a repetition sample examined the items and scales. The item selection and scale validation were conducted in the tradition of classical test theory using: a) latest programming for point and interval estimation of item difficulty, item-remainder correlations, and composite reliability if an item were removed; b) First order CFA; and c) Second Order CFA. All analyses were accomplished with LISREL Version 8.72, which furnishes full information maximum-likelihood estimation for missing data for analyzed ordinal variables that use all obtainable data on all items.

Following the classical item approach, the CFA model was fitted to the data for each presented scale. The focus here was to raise a model with a series of items having maximum internal consistency, other things being equal (e.g., item difficulty). Internal consistency was defined as the model having an acceptable fit to the data (Ping, 2004). The consistency of individual scales in a multi-scale list needs to be specifically significant when these lists are to be used for program evaluation where unsuspected construct determination is important. This was obtained using measures of item-remainder correlations and, other things being equal, removing items with the lowest anticipation rates, sequential fitting of CFA models, and evaluation of model fit. CFA models were fitted to the data using the weighted least squares mean and variance adjusted (WSLMV) estimator obtainable in LISREL. With this diagonally-weighted least squares approach, solely the diagonal elements of the weight matrix are used in the assessment while the full weight matrix is employed to calculate standard errors and χ^2^ (Hancock and Mueller, 2013). 

Non-standardized and standardized factor loadings, a measure of the variance in the measured variable clarify by the latent variable (R2), and related standard errors are providing in LISREL 8.72 concurrently with fit statistics (χ^2^, CFI – Comparative Fit Index, GFI- Goodness Fit Index, AGFI- Adjusted Goodness Fit Index, RMSEA – Root Mean Square Error of Approximation, and SRMR-Standard Root Mean Square Residual. Indicative threshold values for the tests of ‘close fit’ used in this identification were CFI>0.95; GFI>0.90; AGFI>0.90; RMSEA<0.06 and SRMR<0.08) while a value of <0.08 for the RMSEA was taken to demonstrate an ‘appropriate’ fit (Bollen and Long, 1993; Yu, 2002; Hoyle, 2012). LISREL also gives statistics that can be used to serve model modification by recommending fixed parameters (e.g., in the case of single-factor models, correlations among residual variances) that might be liberally measured. In LISREL, these statistics comprise standardized residuals, modification indices (MIs) and participatory change in a parameter if the modification is included in the model (Standardized Expected Parameter Change – SEPC).

## Results

*Sample characteristics*

A total of 400 Thai people completed the questionnaire (response rate: 100%), in the age range of 50 to 65 years (Mean=57.4, SD=4.616). The majority of participants (85.75%) had not achieved more than a primary school education and approximately 85% had occupations in agriculture. Demographic characteristics are summarized in Table 1.

*Construct validity*

Unweighted least squares confirmatory factor analysis was employed to assess the CRC-LS measurement model fit, and the resulting standardized loadings are provided in Table 2.

The first order confirmatory factor analysis revealed the six factor structure of the CRC-LS model to fit the data adequately for each domain as follows: 1) Knowledge and understanding of colorectal cancer screening domain had a good construct (Chi-squared/degree of freedom = 1.012, p = 0.445, CFI = 1.00, GFI = 0.97, AGFI = 0.93, RMSEA = 0.005 and SRMR = 0.032); 2) Accessibility of information and service about colorectal cancer screening domain had a good construct (Chi-squared/degree of freedom = 1.323, p = 0.178, CFI = 1.00, GFI = 0.99, AGFI = 0.96, RMSEA = 0.028 and SRMR = 0.026); 3) Communication skill with health care providers domain had a good construct (Chi-squared/degree of freedom = 1.098, p = 0.345, CFI = 1.00, GFI = 0.99, AGFI = 0.97, RMSEA = 0.016 and SRMR = 0.013); 4) Self-management in the prevention of colorectal cancer domain had a good construct (Chi-squared/degree of freedom = 1.033, p = 0.410, CFI = 1.00, GFI = 0.99, AGFI = 0.98, RMSEA = 0.009 and SRMR = 0.016); 5) Media literacy of colorectal cancer screening information domain had a good construct (Chi-squared/degree of freedom = 1.790, p = 0.167, CFI = 1.00, GFI = 1.00, AGFI = 0.97, RMSEA = 0.045 and SRMR = 0.012); and 6) Decision skills for Colorectal Cancer Screening domain had a good construct (Chi-squared/degree of freedom = 1.465, p = 0.164, CFI = 1.00, GFI = 0.99, AGFI = 0.97, RMSEA = 0.034 and SRMR = 0.027). All items significantly loaded on their respective factors and the resulting fit statistics are provided in Table 3.

Second order confirmatory factor analysis demonstrated that the initial six-factor model of the CRC-LS (69 items) did not fit. Twelve items (K7, K8, K12, K17, K25, ac7, ac8, ac9, ac10, sm6, ds1, ds5) were stepwise removed until the goodness of fit indicators of the final model, consisting of 57 items (6- factor model), demonstrated a fit. The goodness of fit indices indicated that the final model had a good construct (Chi-squared/degree of freedom = 1.079, p = 0.061, CFI = 1.00, GFI = 0.93, AGFI = 0.91, RMSEA = 0.014 and SRMR = 0.036) as shown in Table 4. The KMO was 0.903, and Bartlett’s test of sphericity was significant (p <0.001) indicating reasonable adequacy of the data for factor analysis.

The interfactor correlation matrix revealed significant association between several subscales of the CRC-LS instrument. The knowledge and understanding of colorectal cancer screening subscale was positively associated with Four other subscales, such as Accessibility of information and service about colorectal cancer screening subscale, communication skill with health care providers subscale, self-management in the prevention of colorectal cancer subscale and Media literacy of colorectal cancer screening information subscale. The communication skill with health care providers subscale was negatively associated with decision skills for Colorectal Cancer Screening subscale. Although some of the other interfactor correlations were statistically significant, the small magnitude of these correlations suggests the analysis was overpowered. Details of the correlation analysis of the CRC-LS subscales are presented in Table 5.

*Reliability*

The reliability of the CRC-LS was evaluated for internal consistency (Cronbach’s alpha). The alpha value of 0.948 indicated sufficiently high reliability to provide confidence interpreting the score overall. The correlation item total score range was 0.70 to 0.93.

## Discussion

Establishing comprehensive measures of colorectal cancer screening literacy will play an essential role in developing early-detection programs for colorectal cancer in middle-income countries such as Thailand.. To the best of our knowledge, this is the first instrument developed to evaluate colorectal cancer screening literacy in Thai people. Our questionnaire features strong internal consistency reliability and most of the items included strongly parallel with hypothesized constructs. The CRC-LS was designed to be a self-administered questionnaire that is easy to use and will allow researchers and practitioners to obtain a better perception of colorectal cancer screening literacy in Thai people for both general observational studies and for measuring the efficacy of colorectal cancer screening literacy interventions.

Our study demonstrated that the CRC-LS relevant principle domains for the assessment of colorectal cancer screening literacy include knowledge and understanding of colorectal cancer screening, accessibility of information and service about colorectal cancer screening, communication skills with health care providers, self-management in the prevention of colorectal cancer, media literacy for colorectal cancer screening information and decision skills for colorectal cancer screening. A few domains of the CRC-LS were consistent with those identified in earlier studies, including knowledge and understanding of colorectal cancer screening, accessibility of information and service about colorectal cancer screening (Van der Heide et al., 2015; Peterson et al., 2007; Woudstra et al., 2017; Kobayashi et al., 2014; Arnold et al., 2017). Apart from these normally used domains, we identified novel domains not included in a majority of existing instruments. In particular, communication skills with health care providers, self-management in the prevention of colorectal cancer, media literacy for colorectal cancer screening information and decision skills for colorectal cancer screening appeared important. We propose the addition of these domains to an instrument assessing colorectal cancer literacy will give a better understanding of colorectal cancer screening literacy. For all domains of the CRC-LS, we focused on measuring individual literacy relevant to colorectal cancer screening behavior (Ojinnaka et al., 2015; Wilson et al., 2010).

A large number of existing instruments focus principally on colorectal cancer screening behavior as an indicator of colorectal cancer literacy. However, several studies have advocated health literacy as an important component (Sentell et al., 2013; Woudstra et al., 2017; Essink-Bot et al., 2016; Brittain et al., 2016). In this context it should be stressed that the CRC-LS presented good internal consistency (overall 0.95, and its subscales ranged from 0.70 to 0.93). This is a prerequisite for assessing existing levels of health literacy to plan comprehensive health programs of colorectal cancer, such as early detection and treatment of colorectal cancer in Thailand. A previous study on colorectal cancer literacy was trialed and developed (e.g., the ACCL) (Pendlimari et al., 2012) especially for the US health system and actually refers to specific programs (e.g., colonoscopy) offered in this health setting. The ACCL is unlikely to be appropriate outside the US. Our findings are more likely to be useful for measuring colorectal cancer literacy in the Thai population.

CFA, used to confirm a construct validity of instruments, has become a popular method used for assessment of factor structure to give the basis for convergent and discriminant validity of theoretical constructs (Byrne, 2010). Most items were removed because of low factor loading and important overlapping (high MI), allowing improvement of the fit indices of the model. Nevertheless, the panel of this study decided for investigation of the items before they were removed as they might represent significant and meaningful constructs. A few limitations were discovered. This study was validated among Thai people in a Northeastern Thailand and the results may also be usable in other areas in Thailand. However, because a large number of items deeded to be removed during the study, the CRC-LS should be managed with particular care until cross-validation studies can be undertaken in other areas. Another limitation was that correlation of the CRC-LS with other instruments was not possible. Thus, we suggest further research to validate the Thai version of the CRC-LS in other areas and to correlate it with other instruments.

This study also had some important strengths. First, we conducted a thorough literature review and collected an extensive pool of items which were subsequently assessed by a panel of experts. Second, our questionnaire was developed for the general population and most subjects were over 50-y of age, in contrast to the ACCL (Pendlimari et al., 2012), currently the most widely used measure of functional health literacy. Third, a large part of colorectal cancer screening literacy instrument validations have had important methodological limitations, or fallen well short of full validation. For example, factor analysis has not been complete for every trial to justify or validate their reported domains. Our study included a suitable Confirmatory Factor Analysis and we plan to additionally employ the test-retest method in a future study.

In conclusion, the described CRC-LS enjoys good psychometric properties to assess colorectal cancer literacy among Thai people and should contribute to appropriate educational programs for improved understanding. Future studies should emphasize further criterion-related validation, including attention to the test-retest method.
